# Endometriosis as a Comorbid Condition in Chronic Fatigue Syndrome (CFS): Secondary Analysis of Data From a CFS Case-Control Study

**DOI:** 10.3389/fped.2019.00195

**Published:** 2019-05-21

**Authors:** Roumiana S. Boneva, Jin-Mann S. Lin, Friedrich Wieser, Urs M. Nater, Beate Ditzen, Robert N. Taylor, Elizabeth R. Unger

**Affiliations:** ^1^Division of High-Consequence Pathogens and Pathology, National Center for Emerging and Zoonotic Infectious Diseases, Centers for Disease Control and Prevention, Atlanta, GA, United States; ^2^Department of Gynecology and Obstetrics, Emory University School of Medicine, Atlanta, GA, United States; ^3^Department of Psychiatry and Behavioral Sciences, Emory University School of Medicine, Atlanta, GA, United States

**Keywords:** endometriosis, chronic fatigue syndrome, chronic pelvic pain, menopause, hysterectomy, sleep, cortisol, inflammatory markers

## Abstract

**Background:** Endometriosis (EM) is a recognized co-morbid condition in women with chronic fatigue syndrome (CFS). This analysis evaluates the impact of EM on the health of women with CFS by comparing selected health characteristics and laboratory parameters in women with CFS with and without EM (CFS+EM and CFS-only).

**Methods:** This secondary analysis included all 36 women with CFS from a cross-sectional study of CFS in Wichita, KS, conducted between 2002 and 2003. The health characteristics and laboratory parameters of interest included functioning, fatigue, CFS-related symptoms, gynecologic history, routine laboratory parameters, inflammatory markers, cortisol levels, allostatic load, and sleep parameters (overnight polysomnography). We used parametric or non-parametric tests to compare group differences in the selected health characteristics and laboratory parameters. For examining the association between EM and variables of interest, logistic regression models were performed and odds ratios (OR) with 95% confidence intervals (CI) were reported for the magnitude of associations. Statistical significance was set at 0.05 (two-sided).

**Results:** The mean age of this study sample was 50.9 years. Of women with CFS, 36.1% reported having EM. Age and body mass index (BMI) did not differ between CFS+EM and CFS-only groups. When examining the impact of EM, compared to women with CFS-only, women with both CFS and EM were more likely to report chronic pelvic pain [OR = 9.00 (95% CI, 1.47–55.25)] and hysterectomy [OR = 10.3 (1.82–58.39)], had more CFS symptoms (6.8 ± 0.3 vs. 5.5 ± 0.3, *p* = 0.02), younger mean age at menopause onset (36.4 ± 3.0 vs. 47.0 ± 2.7 years, *p* = 0.03), higher mean number of obstructive apnea episodes per hour (20.3 vs. 4.4, *p* = 0.05) and reported more negative life events (15.8 vs. 4.4, *p* = 0.05). Other parameters did not differ significantly between the two groups.

**Conclusions:** We found more than a third of women with CFS reported endometriosis as a comorbid condition. The endometriosis comorbidity was associated with chronic pelvic pain, earlier menopause, hysterectomy, and more CFS-related symptoms. However, endometriosis in women with CFS did not appear to further impact functioning, fatigue, inflammatory markers, or other laboratory parameters. Further investigations including younger women are warranted.

## Introduction

Chronic fatigue syndrome (CFS), also referred to as myalgic encephalomyelitis (ME) or ME/CFS, is a serious chronic condition characterized by significant impairment in activity levels due to profound fatigue, worsening symptoms after seemingly minimal physical, or mental exertion, sleep problems, as well as difficulties with memory and concentration or orthostatic intolerance (1). Patients with ME/CFS also frequently experience chronic joint and muscle pain. Conditions with chronic pain as a major symptom, such as ME/CFS, endometriosis (EM), fibromyalgia, interstitial cystitis/bladder pain, irritable bowel syndrome, temporomandibular joint syndrome, and chronic migraines, have been termed chronic overlapping pain conditions. There is evidence that persons with one of these conditions are more likely to have another one as a co-morbidity (2, 3). We conducted this analysis to examine the functional impact of EM as a co-morbid condition in women with CFS.

We focused on EM as a comorbidity for several reasons. EM is an estrogen dependent inflammatory disease, which affects 5–10% of women of reproductive age (4, 5); 0.5 to 5% of fertile women and 24–40% of infertile women (6–8). In a survey of women with EM, about 20% reported one or more co-morbidities such as CFS, autoimmune diseases, migraine, and other chronic pain syndromes (9). That survey estimated that, compared to women in the general population, those with EM were eight times more likely to have CFS. Similarly, among women with CFS, EM is a common comorbidity. In a study of CFS that focused on reproductive history risk factors, 19% of women with CFS reported EM whereas only 8% of women without CFS reported EM (10). Reports also show that CFS and EM share a variety of abnormalities and “risk factors.” Changes in daily cortisol secretion have been reported in persons with EM (11, 12) as well as in those with CFS (13). Stress has been implicated in the pathogenesis of EM and its symptoms (9, 14, 15) as well as in chronic pain syndromes, such as fibromyalgia and CFS (16, 17). Allostatic load, a measure of life-long-stress, has been shown to be higher in women with CFS (18).

In this report, we sought to examine the impact of EM on CFS. More specifically, we compare women with CFS and EM (termed “CFS+EM”) and women with CFS only (termed “CFS-only”) in regard to health and function scores, fatigue scores, number of CFS symptoms, select variables from the gynecological history, psychosocial and stress variables, and select laboratory parameters.

## Methods

### Data Source and Study Sample

Data were derived from participants in a 2-day in-hospital case-control study (the source study) of CFS conducted between 2002 and 2003. Participants were previously identified in the Wichita (Kansas, USA) 4-year longitudinal population-based surveillance study of CFS (18). The source study adhered to U.S. Department of Health and Human Services human experimentation guidelines and received Institutional Review Board approval from CDC and Abt associates. All participants gave written informed consent. Demographic data were collected during a computer assisted telephone interview and confirmed at the clinic. Participants underwent a comprehensive in-hospital clinical evaluation over 2 days. The study used the operationalized 1994 CFS case definition (19, 20). Participants who had medical or psychiatric conditions that could explain their symptoms were excluded from the CFS classification. In the source study, 43 persons were diagnosed as having CFS (18, 20); 36 of them were women and were all included in this secondary analysis. Body mass index (BMI) was calculated from height and weight measured at the clinic at the time of physical exam: BMI = weight [kg]/ height^2^ [m^2^].

### Patient-Reported Outcomes

We assessed functional health and well-being with the Medical Outcomes Survey short form-36 version 2 (SF-36 v2) (21, 22) and fatigue with the 20-item multi-dimensional fatigue inventory (MFI-20) (23). We used the CDC Symptom Inventory (CDC-SI) to evaluate the presence, frequency, and severity/intensity of all CFS case-defining symptoms, with SI score calculated as the product of the frequency and intensity/severity of symptoms (24–26). The higher the scores of MFI-20 and CDC-SI scales, the worse the fatigue/symptom severity; in contrast–lower SF-36 scores reflect worse functional health.

We collected data on gynecologic conditions and surgeries using a previously described short, structured gynecologic history questionnaire (27). The questionnaire included questions relating to endometriosis (“Have you ever suffered from endometriosis?”), chronic pelvic pain (“In the past 6 months have you experienced lower abdominal or pelvic pain that is unrelated to your menstrual periods?”) and menopause (menopausal yes or no; if yes, age their periods stopped). Women were also asked whether they had had their uterus and/or ovaries removed.

We assessed stress with the Short Form of the Childhood Trauma Questionnaire (CTQ) for severity of different types of childhood trauma (28), the Perceived Stress Scale (PSS) (29) for an index of chronic stress or strain, and coping with these stresses, and the Life Experiences Survey (LES) (30) for acute and chronic life stresses. We used the self-administered Spielberger State-Trait Anxiety Inventory (STAI) to measures core symptoms of anxiety as a general trait and as a current state based on responses to 40 items (31). We used the Self Rating Depression Scale (SDS), a 20-item questionnaire, to measure core symptoms of depression on a 4-point Likert scale (32). Subjects were screened for posttraumatic stress disorder (PTSD) based on the self-administered Davidson Trauma Scale (DTS) (33).

### Sleep Study

Polysomnography parameters were derived from the overnight polysomnography recordings performed during the second in-clinic night and aggregate results for CFS cases and controls along with detailed methods have been previously reported (34, 35). In brief, polysomnography was performed in the period 10:00 p.m. (when lights were turned out) until 7:00 a.m. the next day. We used data from women with CFS (with or without EM) and included variables of total sleep time, respiratory disturbance index (RDI) scores, snore index, obstructive sleep apnea (OSA) scores, sleep efficiency, and wake time.

### Laboratory Tests of Biological Specimens

Sample collection and testing have been described (36). Complete blood counts (CBC), routine blood chemistry, serum cortisol, catecholamines, and inflammatory markers (C-reactive protein [CRP], the pro-inflammatory cytokines interleukin 6 [IL-6], and tumor necrosis factor alpha [TNF-α]) were measured from fasting blood samples obtained at 7:00 a.m. In addition to serum cortisol, 24 h urinary cortisol was also measured.

### Allostatic Load Index (ALI)

Allostatic load is the body's cumulative “wear and tear” due to repeated cycles of adaptation to stress (37) and could be measured by a composite score called allostatic load index (ALI). This index included 11 components representing metabolic and cardiovascular parameters, inflammatory response parameters, hypothalamus-pituitary-adrenal axis activity parameters, and measures of sympathetic nervous system (SNS) activity. Aggregate data on allostatic load for all the CFS cases and their controls have been previously reported (38).

In this report, comparisons of health parameters focus on women with “CFS+EM” vs. women with “CFS-only.” Data on controls with endometriosis (“EM-only”) and controls without EM are available in the appended material (which includes tables with results across four groups: “CFS+EM,” “CFS-only,” “Controls with EM,” and “Controls without EM”). We present some of those data in the main text when relevant.

### Statistical Analyses

We used the Chi-square (or Fisher's exact test, when indicated) to examine the independence of categorical variables and analysis of variance to test mean differences between the two groups. Mean and Standard Error of Mean (SEM) were used to summarize the results. Cortisol values were log-transformed to obtain normal or near-normal data distribution. Where applicable, non-parametric tests were used for comparing non-normally distributed continuous variables. In addition, we used logistic regression to calculate odds ratios (OR) with 95% confidence intervals (CI) as a measure of association between selected health characteristics and study group. For variables known to be associated with BMI, we calculated respectively adjusted ORs. Statistical significance was set at *p* < 0.05.

## Results

The demographic data for women included in this analysis are presented in Table 1. The women's mean (SEM) age was 51.1 (1.0) years, median−52.5 years, range 27–69 years (with 61% being 50 or older). Ninety four percent were white, 72.6% were employed, 55.9% had a household income of over $40,000 and over two-thirds had more than high school education (Table 1). Endometriosis was reported significantly more frequently by the CFS group−36% (13 of 36) than by the well, non-fatigued controls−17% (8 of 48), *p* = 0.04 (see Tables 1S, 2S).

**Table 1:** Demographic characteristic for the sample of 36 women with chronic fatigue syndrome (CFS cases) in the case-control study of chronic fatigue syndrome, Wichita, USA, 2002–2003.

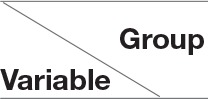	**All CFS (*n* = 36)**
Mean age (SEM)	50.9 (1.5)
**Age Group**	
18–29	1 (2.8%)
30–39	4 (11.1%)
40–49	10 (27.8%)
50 and older	21 (58.3%)
**Race**	
White	32 (88.9%)
Other (Black and Native American)	4 (11.1%)
**Education**	
High school graduate^(c)^	14 (38.9%)
Associate degree, some college, or college degree	22 (61.1%)
**Income ($/Year)**	
=<20,000	11 (30.6%)
20,000–40,000	13 (36.1%)
Over 40,000	9 (25.0%)
Missing	3 (12.5%)

*All values are numbers and (%) except for age, which is shown as mean*.

*SEM, standard error of the mean*.

(c) *, includes one participant who had some high school education but no diploma*.

The “CFS+EM” and “CFS-only” subgroups did not differ significantly in mean age or mean BMI (Table 2). In both subgroups most women reported gradual onset of CFS and there was no significant difference in mean duration of the CFS illness (~17 years) (Table 2). Women with CFS+EM reported post-exertional malaise more frequently (92.3%) than women with CFS-only (78.3%) but this difference was not statistically significant. There were no significant differences in the means of the SF-36 subscale scores and the MFI-20 scores (Table 2). However, compared to CFS-only, the CFS+EM group had a significantly greater number of CFS symptoms from the symptom inventory (SI): 6.8 ± 0.3 (mean ± SEM) vs. 5.5 ± 0.3, *p* = 0.02; the total CFS SI score was also higher (51.4 ± 5.7 vs. 43.0 ± 4.3) but not significantly different, *p* = 0.30.

**Table 2:** Comparison of select characteristics in women with CFS by subgroup—with and without endometriosis comorbidity.

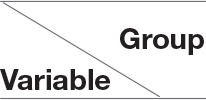	**All CFS (*n* = 36)**	**CFS+EM (*n* = 13)**	**CFS-only (*n* = 23)**	***p***
Age	50.9 (1.5)	54.0 (1.8)	49.1 (2.1)	0.39
BMI	29.5 (0.7)	28.6 (1.0)	30.0 (1.0)	0.29
Mean duration of CFS illness (years)	17.0 (2.7)	16.8 (3.3)	17.1 (2.3)	0.93
Onset of CFS illness^(a)^				0.73
Sudden, *n (%)*	6 (16.7%)	2 (15.4%)	4 (17.4%)	
Gradual, *n (%)*	29 (80.6%)	11 (84.6%)	18 (78.3%)	
Missing, *n (%)*	1 (2.7%)	—	1 (4.3%)	
Presence of post-exertional malaise				0.28
Yes, *n (%)*	30 (83.3%)	12 (92.3%)	18 (78.3%)	
No, *n (%)*	6 (16.7%)	1 (7.7%)	5 (21.7%)	
**SF-36 Subscales (Range 0–100)^(b)^**
General health	51.6 (3.5)	53.9 (5.7)	50.3 (4.6)	0.64
Mental health	66.0 (3.3)	68.6 (6.5)	64.5 (3.6)	0.55
Physical functioning	50.4 (3.6)	46.9 (5.4)	52.4 (4.7)	0.47
Role emotional	55.6 (7.0)	64.1 (11.6)	50.7 (8.9)	0.37
Role physical	18.8 (5.0)	11.5 (7.8)	22.8 (6.5)	0.29
Social functioning	49.0 (3.9)	49.0 (7.9)	48.9 (4.2)	0.99
Vitality	17.8 (2.1)	19.6 (4.0)	16.7 (2.3)	0.51
Bodily pain	40.1 (2.7)	37.0 (3.8)	41.8 (3.7)	0.40
**MFI-20 Subscales (Range 4–20)^(c)^**
General fatigue	17.7 (0.3)	17.1 (0.4)	17.9 (0.4)	0.74
Physical fatigue	14.4 (0.5)	13.8 (0.9)	14.7 (0.6)	0.44
Mental fatigue	14.1 (0.7)	12.8 (1.2)	14.9 (0.8)	0.17
Reduced activity	14.8 (0.6)	14.5 (0.8)	15.0 (0.8)	0.67
Reduced motivation	12.3 (0.7)	12.2 (1.1)	12.3 (0.9)	0.98
**CDC Symptom Inventory**				
Number of CFS symptoms	5.9 (0.2)	6.8 (0.3)	5.5 (0.3)	0.02
Symptom Inventory Score	46.0 (3.4)	51.4 (5.7)	43.0 (4.3)	0.24
**Gynecologic Variables**				
Pelvic pain^(d)^, *n* (%)	8 (22.2%)	6 (46.2%)	2 (8.7%)	0.02
Hysterectomy, *n* (%)	19 (52.8%)	11 (84.6%)	8 (34.8%)	<0.01
Postmenopausal, *n* (%)	25 (69.4%)	12 (92.3%)	13 (56.5%)	0.03
Mean age at menopause onset	41.7 (2.3) (*n* = 20)	36.4 (3.0) (*n* = 10)	47.0 (2.7) (*n* = 10)	0.03
Hysterectomy in the subset of post-menopausal women only, *n* (%)	19/25 (76%)	11/12 (91.7%)	8/13 (61.5%)	0.16

CFS, chronic fatigue syndrome; EM, endometriosis; CFS+EM, women who had CFS and endometriosis; CFS-only, women who had CFS but not endometriosis

*Values are shown as mean (SEM, standard error of the mean) or number (percentage), n (%)*.

(a) *One missing response in the CFS-only group*.

(b) *Lower score indicates worse health status/more disability*.

(c) *Higher score indicates more fatigue*.

(d) *Non-menstrual, chronic pelvic or lower abdominal pain*.

### Gynecologic Characteristics (Table 2)

Non-menstrual, chronic pelvic, or lower abdominal pain was reported significantly more frequently by women with CFS+ EM (46.2%) than the CFS-only group (8.7%), *p* = 0.02; OR = 9.00 (95% CI, 1.47–55.25). Compared to CFS-only, women with CFS+EM were 9 times as likely to be menopausal, OR = 9.23 (1.02–83.33), BMI adjusted OR = 10.79 (1.13–103.11). Notably, among menopausal women, the mean age at menopause onset in the CFS+EM group was 36.4 ± 3.0 years—a decade earlier than in the CFS-only group (47.0 ± 2.7), *p* = 0.03. Hysterectomy rates were significantly higher in the CFS+EM group than the CFS-only group (84.6 vs. 34.8%), OR = 10.31 (95% CI, 1.82–58.39), BMI adjusted OR = 16.13 (2.30–113.23), *p* = 0.005. Of the 36 women with CFS, 8 (22%) reported pelvic pain, 75% of whom also reported endometriosis (Table 2S).

### Psychometric Characteristics (Table 3)

The mean scores for negative life events were significantly higher in women with CFS+EM than in the CFS-only group (15.8 vs. 7.1, *p* = 0.049); the total Life Events Score (LES) was also higher in CFS+EM (19.0 vs. 12.3) but not significantly different. All other scores were similar in the two groups.

**Table 3:** Comparisons of psychometric variables in women with CFS by subgroup—with and without endometriosis.

**Variables**	**CFS+EM (*n* = 13)**	**CFS-only (*n* = 23)**	***p***
	**Mean (SEM)**		
**CTQ Scores**
Emotional abuse	10.8 (1.4)	10.9 (1.4)	0.95
Physical abuse	8.1 (1.2)	7.8 (0.7)	0.85
Sexual abuse	7.5 (0.7)	8.9 (1.4)	0.47
Emotional neglect	12.0 (1.5)	11.7 (1.3)	0.90
Physical neglect	7.9 (1.0)	6.6 (0.5)	0.18
Total CTQ score	46.3 (4.7)	46.0 (4.5)	0.97
**PSS Score** (total)	16.4 (2.1)	17.7 (1.4)	0.58
**LES**			
Positive events	3.2 (1.1)	5.2 (1.2)	0.28
Negative events^*^	15.8 (5.6)	7.1 (1.5)	0.05
Overall LES change	19.0 (5.8)	12.3 (2.1)	0.20
**SDS index**	54.5 (2.0)	55.4 (1.9)	0.77
**STAI**			
Trait	41.1 (3.1)	43.3 (2.3)	0.58
State	38.2 (12.3)	37.7 (2.3)	0.90
**DTS Score** (total)	29.1 (7.5)	30.1 (4.8)	0.90

*CTQ, childhood trauma questionnaire; PSS, perceived stress scale; LES, life experiences survey*,

* *Negative events are presented in absolute value; SDS, self-rating depression scale; STAI, state-trait anxiety inventory; DTS, Davidson trauma scale*.

### Laboratory Parameters (Table 4)

Blood counts and blood chemistry were all within normal limits but the CFS+EM group had slightly higher hemoglobin and hematocrit than the CFS-only group, *p* ≤ 0.05. Inflammatory markers—CRP, IL-6, and TNF-alpha—were not elevated in either subgroup, and TNF-alpha was lower in CFS+EM group than in the CFS group. Serum cortisol, 24-h urinary cortisol, and salivary cortisol levels (appended material) did not differ significantly.

**Table 4:** Comparisons of mean values for selected laboratory parameters and allostatic load index in women with CFS, by subgroup—with and without endometriosis comorbidity.

**Parameter**	**CFS+EM** **(*n* = 13)**	**CFS-only** **(*n* = 23)**	***p***
Hemoglobin	13.8 (0.2)	13.3 (0.1)	0.04
Hematocrit	40.6 (0.6)	39.3 (0.4)	0.05
Red blood cells	4.5 (0.1)	4.5 (0.0)	0.63
WBC	7.5 (0.6)	7.3 (0.3)	0.74
Granulocytes	4.3 (0.4)	4.1 (0.3)	0.76
Lymphocytes	2.4 (0.3)	2.4 (0.2)	0.94
**Protein**			
Total protein	7.4 (0.1)	7.3 (0.1)	0.65
Albumin	3.7 (0.1)	3.6 (0.0)	0.28
**Electrolytes**			
Sodium	140.1 (0.5)	139.6 (0.4)	0.50
Potassium	3.8 (0.1)	3.9 (0.1)	0.58
Calcium	9.0 (0.1)	9.0 (0.1)	0.96
Alkaline phosphatase	92.5 (6.0)	99.4 (7.8)	0.55
Carbon dioxide	27.0 (0.3)	26.1 (0.5)	0.25
Anion gap	9.8 (0.4)	9.8 (0.4)	0.92
**Select inflammatory markers**			
High sensitivity CRP	4.5 (1.2)	5.2 (0.9)^a^	0.46^*^
Interleukin-6 (IL-6)	2.3 (0.3)	2.7 (0.3)^a^	0.34^*^
TNF-alpha	2.1 (0.1)	3.1 (0.4)^a^	0.02^*^
**Cortisol**			
Mean serum free cortisol	19.9 (1.3)	17.1 (1.3)	0.12^*^
Urinary free cortisol/24 h	20.2 (3.5)	17.6 (2.6)	0.53
Allostatic index score	2.9 (0.5)^b^	3.0 (0.4)^c^	0.86

*CFS, chronic fatigue syndrome; EM, endometriosis; CFS+EM, women who had CFS and endometriosis; CFS-only, women who had CFS but not endometriosis; CRP, C-reactive protein; IL-6, interleukin 6; TNF, tumor necrosis factor*.

a *Values available for 20 subjects*.

b *Values available for 8 subjects*.

c *Values available for 14 subjects*.

* *Kruskal-Wallis test*.

### Sleep Study: Polysomnography Parameters (Table 5)

The total sleep time per night was about 20 min shorter in the CFS+EM group than in the CFS-only group (394 ± 15.8 vs. 414.7 ± 9.0), *p* = 0.30. The CFS+EM had higher mean scores for OSA episodes (20.3 ± 11.3 events/h, i.e., within the moderate severity range) than the CFS-only group (4.0 ± 2.3), *p* = 0.12 by Kruskal-Wallis test, *p* = 0.05 after adjusting for BMI. The other sleep parameters did not differ between the two groups.

**Table 5:** Sleep characteristics of the women with chronic fatigue syndrome, by subgroup—with and without endometriosis.

**Sleep variable**	**CFS+EM** **(*n* = 13)**	**CFS-only** **(*n* = 23)**	***p*** ***(BMI adjusted)***
	**Mean (SEM)^a^**		
Total sleep time (minutes)	394.5 (15.8)	414.7 (9.0)	0.30
Respiratory disturbance index	8.2 (3.1)	6.5 (2.3)	0.31 (0.16^*^)
Obstructive apnea (episodes per hour)	20.3 (11.3)	4.4 (2.3)	0.05 (0.12^*^)
Snore index	6.5 (3.3)	7.1 (1.7)	0.70
Latency to sleep onset (minutes)	22.8 (6.4)	20.7 (4.3)	0.90
Mean sleep latency (minutes)	10.5 (1.4)	8.9 (1.1)	0.49
Rapid eye movement (REM) sleep (as a proportion of total sleep time)	0.21 (0.0)	0.24 (0.0)	0.45
Sleep efficiency (as a proportion of total sleep time)	0.9 (0.0)	0.9 (0.0)	0.41
Wake (as a proportion of total sleep time)	0.1 (0.0)	0.1 (0.0)	0.41

*CFS+EM, chronic fatigue syndrome and endometriosis comorbidity; CFS-only, chronic fatigue syndrome without endometriosis*.

* Kruskal–Wallis test (cannot be adjusted for BMI)

a *Means are the estimated mean values obtained with BMI included in the generalized linear model*.

## Discussion

In this study, which used a convenience sample from a population-based study of CFS in Wichita, KS, we confirmed a significantly higher prevalence of EM in women with CFS (36%) than in controls without CFS (17%). In this middle-aged group of women with CFS, comorbid endometriosis was associated with a higher number of CFS symptoms, higher prevalence of chronic pelvic pain, higher rates of hysterectomy and menopause and, most notably, with a decade-earlier menopause onset than in women with CFS-only. However, the endometriosis comorbidity in women with CFS was not associated with significantly worse functioning (SF-36 subscales), fatigue (MFI subscales), or laboratory parameters. As a group, women with CFS and EM reported, on average, one more CFS case-defining symptom and had a higher composite symptom inventory score than women with CFS-only. A higher proportion of women with CFS+EM reported post-exertional malaise. Although the latter two differences were not statistically significant, they may be clinically relevant and may reflect a health impact not otherwise captured by instruments such as SF-36 or MFI-20.

### Gynecologic Characteristics

Nearly half (46.2%) of the women with CFS+EM reported chronic pelvic or abdominal pain unrelated to menstrual periods; this is similar to the frequency reported in other studies of EM (39, 40). However, it is unlikely that active EM could explain this pain in our study as only one of the 13 women in the CFS+EM group was not menopausal and 85% of the women had undergone hysterectomy. It may reflect increased pain sensitivity that has been observed in women with chronic pelvic pain (41). The CFS+EM group had a very early mean age at menopause−36 years. This early menopause was probably surgically induced as 85% of the women with CFS+EM reported hysterectomy. A large study of younger US women with EM (mean age 36 years) found that 20% had already undergone oophorectomy and/or hysterectomy (39). Our findings are also similar to the findings from a study in women with fibromyalgia, which found that women with hysterectomy reported more pain and more symptoms than women with fibromyalgia who had not had hysterectomy (42). It is also possible that some patients with chronic pelvic pain who had never undergone surgical intervention may have had endometriosis, which was otherwise undiagnosed. However, only two of the 23 women with “CFS-only” reported chronic pelvic pain. In this case, we might have underestimated the already strong association between chronic pelvic pain and “CFS + EM.” Many of the women in this study were menopausal, which is a limitation of the study as we cannot answer the question whether the enhanced symptomatology in women with “CFS+EM” is due to active endometriosis, residual effect of endometriosis, or is completely unrelated.

### Sleep Parameters

Sleep problems are one of the main symptoms in CFS (19, 43). In our study women with CFS+EM had a mean of 20.3 obstructive sleep apnea events per hour, which places them in the clinical category of moderately severe obstructive sleep apnea (defined as 15–30 events/h), while the CFS-only group had fewer than 5 events/h. The prevalence of obstructive sleep apnea in women is very low prior to menopause but increases sharply after that (44). The CFS+EM group had been in menopause for a mean of ~18 years (while the CFS-only group had been in menopause for a mean of 2 years). Thus, the higher OSA scores in the CFS+EM group may be a corollary of both the earlier onset and higher prevalence of menopause compared with women in the CFS-only group, but it is not likely to be an effect of obesity as women with CFS+EM had slightly lower BMI (mean 28.6 ± 1.0) than those with CFS only (30.0 ± 1.0) and we also adjusted for BMI in the model. The 20 min/night shorter sleep duration in women with CFS+EM (395 min/night) was not statistically different from that in women with CFS-only (414 min/night) but compared to the average 480 min (8 h) considered normal/optimal for adults, the mean sleep duration of the CFS+EM group was 85 min/night shorter (66 min per night for the CFS-only group). Compared to what would be optimal per week, the CFS+EM group appears to accumulate a weekly sleep deficit of more than 9 h (and CFS-only group—a weekly deficit of over 7 h)—that is, they are missing the equivalent of one night of sleep per week.

### Laboratory Parameters

In this cross-sectional study, endometriosis comorbidity in CFS did not affect negatively the studied laboratory parameters. Higher levels of inflammatory markers such as TNF-alpha have been reported in women with EM (45). In our study inflammatory markers were not higher in the CFS group and, on the contrary, TNF-alpha was even lower. Some authors have found higher levels of serum cortisol in infertile women suffering from advanced endometriosis (46) but in our study sample of middle-aged women endometriosis comorbidity was not associated with significant differences in cortisol levels in serum, or 24-h urinary cortisol excretion. It should be noted, however, that most women in our study were middle-aged, postmenopausal and thus older than the premenopausal women included in other studies of inflammatory markers and cortisol levels in endometriosis.

### Psychological Variables

Although previous studies indicate that childhood stress may be linked to chronic pain syndromes such as fibromyalgia and chronic fatigue syndrome (16, 17), we did not find differences between the two groups in relation to stress experienced during childhood. Further, the groups did not differ in perceived stress during the past month, and there were no differences in indices of psychological well-being, such as depression, anxiety, or posttraumatic stress. We may thus conclude that women with CFS and endometriosis are not in worse mental health than those with CFS only, although they do report having experienced more negative life events.

### Study's Strengths and Limitations

Although this study is based on a relatively small, convenience sample, its major strength is that the original study of fatiguing illness was derived from the general population of a defined geographic area and that, during the 2-day clinical evaluation, participants underwent comprehensive clinical, and laboratory evaluation to rule out conditions not compatible with a CFS diagnosis. The availability of data from the structured gynecologic history questionnaire made it possible to conduct this secondary analysis and evaluate the health impact of endometriosis on CFS. Limitations of the study include: the small proportion of women younger than 40 years (the full impact of EM might not be noted as most women were post-menopausal and EM was likely to be “inactive”), recruitment from a single geographic area (Wichita, KS), and lack of racial/ethnic diversity (94% Caucasian women) limiting generalizability of our findings. In addition, the design of the source study did not include review of medical records to confirm self-reported endometriosis and determine method of diagnosis. However, previous studies show that self-reported endometriosis has fairly good predictive ability for diagnostic confirmation in medical records (47) and a high positive predictive value of self-reported gynecologic conditions and surgeries (48). Another limitation of the study is that we did not have information on comorbidities such as irritable bowel syndrome and interstitial cystitis, which play a role in chronic pelvic pain, and we could not control for these conditions in the analyses. In interpreting the study findings it should be kept in mind that data came from a convenience sample—the source study was not specifically designed to evaluate the effects of endometriosis on health in women with CFS, and used a research case definition (19) that does not require the presence of post-exertional malaise as currently recommended by the 2015 Institute of Medicine report for the clinical diagnosis of ME/CFS (1). Further, the mean age of women in this sample was ~51 years and our findings may not be applicable to younger age groups or generalizable to U.S. women outside this community.

## Conclusions

Our study found that patients with CFS and comorbid EM have more CFS symptoms, higher prevalence of chronic pelvic/lower abdominal pain, higher rates of hysterectomy, and significantly earlier onset of menopause than women with only CFS. We did not identify significant differences in functioning, fatigue scores, or inflammatory markers to be associated with comorbid EM. However, the full impact of EM might not be noted in our sample as most women were post-menopausal and EM was likely to be “inactive.” Further studies that include younger and more racially/ethnically diverse women are warranted.

## Ethics Statement

The source study adhered to U.S. Department of Health and Human Services human experimentation guidelines and received Institutional Review Board approval from CDC and Abt associates. All participants gave written informed consent.

## Author Contributions

RB performed the final analysis, interpreted results, and wrote the manuscript. FW, UN, and RB wrote an earlier version (with a different focus) for which UN, FW, RB, JML, RT, and BD all contributed to the earlier analysis and content. All authors have critically read earlier versions of the manuscript and contributed content. EU has critically read and edited previous and current version and made suggestions for its final focus. All authors have read and approved the final version of the manuscript. All authors have contributed to this manuscript.

### Conflict of Interest Statement

The authors declare that the research was conducted in the absence of any commercial or financial relationships that could be construed as a potential conflict of interest.

## Abbreviations

ALI: allostatic load index
BMI: body mass index [kg/m2]
CFS: chronic fatigue syndrome
CFS+EM: CFS with endometriosis
CFS-only: CFS without endometriosis
CRP: C-reactive protein
CTQ: childhood trauma questionnaire
DTS: Davidson trauma scale
EM: Endometriosis
GLM: Generalized Linear Model
IL-6: interleukin 6
LES: life experiences survey
MFI-20: Multiple fatigue inventory
PSS: perceived stress scale
SF-36: a 36-item short-form of the Medical Outcomes Survey questionnaire
OSA: obstructive sleep apnea
SDS: self-rating depression scale
STAI: state-trait anxiety inventory
TNF: tumor necrosis factor.
