# Supplementary material for: Endometriosis as a Comorbid Condition in Chronic Fatigue Syndrome (CFS): Secondary Analysis of Data From a CFS Case-Control Study
Source: Front Pediatr. 2019 May 21;7:195. doi: 10.3389/fped.2019.00195 (PMC6537603; doi:10.3389/fped.2019.00195)
Supplement: Supplementary file 1 [file Data_Sheet_1.docx]

**Supplemental Material**

**Methods—additional information**

**The presence, frequency, and intensity of all CFS** **case-defining** symptoms and other related symptoms were measured by the CDC Symptom Inventory (SI) questionnaire (ref 24, ref 25--Wagner 2005). The questionnaire queries presence, duration, frequency and intensity/severity of eight CFS-defining symptoms as well as the presence of fatigue, post-exertional malaise and other CFS-related symptoms. The number of symptoms can range from 4 case-defining symptoms (the minimum number to qualify as a CFS case) to 8 (the highest number of CFS-case-defining symptoms). The frequency and severity were graded on a scale of 1 to 4 (most frequent or severe). The sum of the products of the frequency and the intensity/severity of each symptom was used to calculate a SI score. To qualify as a CFS case a person must have at least 4 case-defining symptoms and a SI score of at least 25, in addition to having had a fatiguing illness for 6 or more months.

**Gynecologic conditions and surgeries.** Women responded to a short, structured gynecologic history questionnaire. Aggregate results on gynecologic history of CFS cases and well controls (without CFS) have been previously published (ref 27--Boneva et al, 2011). The following questions in the questionnaire related to endometriosis: “Have you ever been diagnosed with endometriosis?” Chronic pelvic pain was assessed by the answer to the question “In the past 6 month, have you experience lower abdominal or pelvic pain that is unrelated to your menstrual periods?” Women were asked whether they were menopausal and those who responded “yes” to this question, were also asked at what age their periods stopped due to menopause. Women were also asked whether they had any gynecological surgery, and if they responded with yes, they had to answer also if they had had their uterus and/or ovaries removed.

**Psychosocial measures**

**Stress**. We used three scales as subjective measures of stress: the Short Form of the Childhood Trauma Questionnaire (CTQ) (ref. 17--Heim 2009), the Perceived Stress Scale (PSS) (29— Cohen 1983), and the Life Experiences Survey (LES) (30—Sarason1978). The CTQ, which measures the severity of emotional abuse and neglect, physical abuse and neglect, and sexual abuse, scores 25 items on a 5-point scale from 1 (never true) through 5 (very often true). The sum of the responses to five items in each domain forms the subscale score. The PSS consists of 10 items that measure chronic stress during the past four weeks on a five-point scale from “never” to “very often”. The PSS assessed how unpredictable, uncontrollable, and overwhelming participants consider their lives to be. The PSS provides a summary score. The LES asked subjects to rate the impact of 44 life events in the past year as positive (3, 2, 1), negative (-3, -2, -1), or neutral (0). Summing the impact ratings of those events designated as positive by the subject provides a positive change score whereas the summing of the negative impact ratings of those events provides a negative change score. A total change score can be obtained by adding these two change scores, representing the total amount (in absolute value) of rated change (desirable and undesirable).

**Evaluation for anxiety, post-traumatic stress disorder and depression**. We used the self-administered Spielberger State-Trait Anxiety Inventory (STAI) to measures core symptoms of anxiety as a general trait and as a current state based on responses to 40 items (ref 31--Spielberg et al, 1970). To assess subjects for posttraumatic stress disorder (PTSD) we used the self-administered Davidson Trauma Scale (DTS) (ref. 33--Davidson et al, 1997); a total score of 40 or higher indicates clinically relevant PTSD. We used the Self-Rating Depression Scale (SDS), a 20-item questionnaire, to measure core symptoms of depression on a 4-point Likert scale (ref 32--Zung 1965). The scale provides a summary score from which an SDS index score is calculated: SDS Index = (Raw Score/maximum possible points) x 100; an SDS score higher than 50 (or SDS index>62.5) indicates depression.

**Laboratory tests**.

Fasting blood samples were collected at 7:30 am after overnight fasting. Participants remained recumbent for 30 minutes prior to blood collection. Blood was drawn for complete blood counts (CBC), routine blood chemistry, endocrine tests, catecholamines, and inflammatory markers (C-reactive protein [CRP], the pro-inflammatory cytokines interleukin 6 [IL-6], and tumor necrosis factor alpha [TNF-α]). Tests for CBC, blood chemistry, urinalysis and basal levels of plasma catecholamines were performed by Quest Diagnostics (KS, USA). Cytokines were measured using an enzyme-linked immunosorbent assay (ELISA) kit (RayBiotech, GA, USA) and all cytokine assays were performed at the CDC.

**Cortisol levels** were measured in serum and in a sample of 24 hour urine. All cortisol testing was completed by a commercial laboratory (Esoterix Inc. Laboratory Services) on stored samples. Details on laboratory assays conducted to assess endocrine status have been published previously (ref 36--Vernon et al., 2006). All laboratory testing was performed according to standard clinical/laboratory protocols.

The 24-hour urine samples were collected between 7:00 am on day one and 7:00 am on day two of the hospital stay according to laboratory protocol. Upon awakening, the first morning urine was discarded and each portion of urine for the next 24 hours was collected in a container (the last portion being the one after awakening on the next day). During the 24 hour collection the urine was kept in a refrigerator. At completion of collection, the total quantity of urine was recorded and an aliquot of 30 ml of well-mixed 24-hour urine was prepared for testing. Boric acid (0.5g/100ml) was used as a preservative. Specimens were stored at -20ºC and shipped for testing. Free cortisol concentration in urine was measured by RIA with sensitivity of the assay is 0.05 ug/dL, intra-assay coefficient of variance (CV) 4.7-10.7 and inter-assay CV 16%. Total urinary free cortisol was calculated from the measured cortisol concentration and the total amount of 24 hour urine.

**Allostatic load** is a measure of the body’s “wear and tear” in response to stress and adaptation (ref 37--McEwen, 1993). In this study we included 11 components: metabolic (waist to hip ratio), cardiovascular activity (systolic and diastolic blood pressure, serum aldosterone); inflammatory response parameters (plasma levels of IL-6, CRP, and albumin); HPA-axis activity parameters (24-hour urinary cortisol, serum DHEA-S) and measures of sympathetic nervous system (SNS) activity (i.e., plasma norepinephrine, epinephrine). Aggregate results for the CFS group and the control group (i.e., well without CFS) have been reported previously (Maloney et al. 2006). Risk cut-offs for these 11 factors were based on quartile risk levels (i.e., 25th percentile and 75th percentile) determined for the non-fatigued controls in the study, except for blood pressure indices, which were based on clinical reference values (ref 38--Maloney et al. 2006).

**Supplemental tables**

**Table 1S. Demographic characteristic for the sample of 36 women with chronic fatigue syndrome (CFS) by subgroup: with endometriosis (CFS+EM) and without endometriosis (CFS-only)**.

| **Group**  **Variable** | All CFS  (n=36) | CFS+EM  (n=13) | CFS-only  (n=23) | p |
| --- | --- | --- | --- | --- |
| Mean age (SEM) | 50.9 (1.5) | 54.0 (1.8) | 49.1 (2.1) | 0.39 |
| Age group |  |  |  | 0.24, 0.14^(a)^ |
| 18-29 | 1 (2.8%) | 0 | 1 (4.3%) |  |
| 30-39 | 4 (11.1%) | 0 | 4 (17.4%) |  |
| 40-49 | 10 (27.8%) | 3 (23.1) | 7 (30.4%) |  |
| 50 and older | 21 (58.3%) | 10 (76.9%) | 11 (47.8%) |  |
| Race |  |  |  | 0.54^(b)^ |
| White | 32 (88.9%) | 12 (92.3%) | 20 (87.0%) |  |
| Other (Black and Native American) | 4 (11.1%) | 1 (7.7%) | 3 (12.5%) |  |
| Education |  |  |  | 0.31 |
| High school graduate^(c)^ | 14 (38.9%) | 3 (23.1%) | 11 (47.8%) |  |
| Associate degree, some college or college degree | 22 (61.1%) | 10 (76.9%) | 12 (52.2%) |  |
| Income ($/year) |  |  |  | 0.57 |
| =< 20,000 | 11 (30.6%) | 4 (30.8%) | 7 (30.4%) |  |
| 20,000 to 40,000 | 13 (36.1%) | 5 (38.5%) | 8 (34.8%) |  |
| Over 40,000 | 9 (25.0%) | 2 (15.4%) | 7 (30.4%) |  |
| Missing | 3 (12.5%) | 2 (15.4%) | 1 (4.4%) |  |
|  |  |  |  |  |

^(a)^Fisher’s exact test two-tailed p value for age as dichotomous variable: p=0.14 for less than 40 versus 40 and older.

^(b)^Fisher’s exact test two-tailed p

^(c)^Includes one woman with CFS-only who had some high school education but no diploma.

**Table 2S (extended to include well controls). Comparisons of select characteristics for cases with CFS+EM versus CFS-only and for controls with endometriosis versus controls without endometriosis: case-control study of chronic fatigue syndrome in Wichita, Kansas (2002-2003).**

|  | **CFS cases**  (n=36 women) | | | **Well controls**  (n=48) | | |
| --- | --- | --- | --- | --- | --- | --- |
| **Variable** | **CFS+EM**  (n=13) | **CFS-only** (n=23) | **p**    (CFS+EM) versus (CFS-only) | **Con+EM**  (n=8) | **Con without EM** (n=40) | **p**  (Con+EM) versus (Con w/o EM) |
| Age, mean (SEM) | 54.0 (1.8) | 49.1 (2.1) | 0.39 | 54.8 (2.9) | 50.5 (1.4) | 0.61 |
| BMI, mean (SEM) | 28.6 (1.0) | 30.0 (1.0) | 0.29 | 26.6 (1.5) | 29.6 (0.8) | 0.36 |
| **SF-36 subscales** (range 0-100),^ |  |  |  |  |  |  |
| General health | 53.9 (5.7) | 50.3 (4.6) | 0.93 | 83.3 (5.0) | 87.6 (1.9) | 0.91 |
| Vitality | 19.6 (4.0) | 16.7 (2.3) | 0.91 | 75.0 (4.5) | 73.5 (2.5) | 0.99 |
| Bodily Pain | 37.0 (3.8) | 41.8 (3.7) | 0.82 | 79.5 (5.4) | 78.8 (2.0) | 0.99 |
| **MFI-20 Subscales** (range 4-20)* |  |  |  |  |  |  |
| General fatigue | 17.1 (0.4) | 17.9 (0.4) | 0.74 | 8.0 (0.9) | 7.8 (0.4) | 0.99 |
| Physical fatigue | 13.8 (0.9) | 14.7 (0.6) | 0.80 | 6.0 (0.5) | 6.8 (0.4) | 0.88 |
| Mental fatigue | 12.8 (1.2) | 14.9 (0.8) | 0.28 | 6.4 (0.9) | 6.3 (0.4) | 0.99 |
| **Number of CFS symptoms,** mean (SEM) | 6.8 (0.3) | 5.5 (0.3) | **0.02** | 2.3 (0.5) | 1.6 (0.2) | 0.52 |
| **Symptom Inventory Score,** mean (SEM) | 51.4 (5.7) | 43.0 (4.3) | 0.30 | 6.6 (1.8) | 4.3 (0.7) | 0.98 |
| **Gynecologic history** |  |  |  |  |  |  |
| Pelvic pain, n (%) | 6 (46.2%) | 2 (8.7%) | **0.02** | 1 (12.5%) | 0 (0%) | 0.17** |
| Postmenopausal, n (%) | 12 (92.3%) | 13 (56.5%) | **0.03** | 8 (100%) | 27 (67.5%) | 0.09 |
| Age at menopause onset, mean (SEM) | 36.4 (3.0)  (n=10) | 47.0 (2.7)  (n=10) | **0.03** | 50.8 (1.3)  (n=4) | 45.3 (1.7)  (n=24) | 0.63  **0.03**§ |
| Hysterectomy, n (%) | 11 (84.6%) | 8 (34.8%) | **0.006** | 7 (87.5%) | 12 (30%) | 0.004 |

CFS, chronic fatigue syndrome;

EM, endometriosis;

Con, controls;

SEM, standard error of the mean;

Values are mean (SEM) or number, n (%);

This table includes only select subscales from the SF-36 and the MFI-20 subscales. ^ Lower score indicates worse health status/more disability;

*Higher score indicates more fatigue.

** Fisher’s exact test two-tailed p value.

§ p value for comparison of mean age at menopause for each of the control subgroups to the CFS+EM subgroup.
